# Enhanced cytotoxicity of reovirus and radiotherapy in melanoma cells is mediated through increased viral replication and mitochondrial apoptotic signalling

**DOI:** 10.18632/oncotarget.10365

**Published:** 2016-07-01

**Authors:** Gráinne McEntee, Joan N. Kyula, David Mansfield, Henry Smith, Michelle Wilkinson, Claire Gregory, Victoria Roulstone, Matt Coffey, Kevin J. Harrington

**Affiliations:** ^1^ Targeted Therapy Team, the Institute of Cancer Research, London, UK; ^2^ Sarcoma/Melanoma Unit, Department of Academic Surgery, Royal Marsden Hospital NHS Foundation Trust, London, UK; ^3^ Oncolytics Biotech Inc., Calgary, Canada

**Keywords:** reovirus, radiotherapy, melanoma, CUG2, apoptosis

## Abstract

Oncolytic viruses selectively target and replicate in cancer cells, providing us with a unique tool with which to target and kill tumour cells. These viruses come from a diverse range of viral families including reovirus type 3 Dearing (RT3D), a non-pathogenic human double-stranded RNA oncolytic virus, which has been shown to be an effective therapeutic agent, both as a mono-therapy and in combination with traditional chemotherapeutic drugs. This study investigated the interaction between RT3D and radiotherapy in melanoma cell lines with a BRAF mutant, Ras mutant or BRAF/Ras wild type genotype. The data indicates that RT3D combined with radiotherapy significantly increased cytotoxicity relative to either single agent, independent of genotype, both *in vitro* and *in vivo*. The mechanism of enhanced cytotoxicity was dependent on an increase in viral replication, mediated by CUG2 up-regulation and subsequent down-regulation of pPKR and p-eIF2α, leading to the activation of mitochondrial apoptotic signalling resulting in increased cell death.

## INTRODUCTION

Oncolytic viruses selectively target and kill cancer cells, leaving untransformed cells unaffected. This unique ability has been exploited in recent years, with a number of viruses including reovirus type 3 Dearing (RT3D) [1], Newcastle disease virus [2], herpes simplex virus type I [3] and coxsackievirus A21 [4] having been extensively studied for their therapeutic potential.

Reovirus has been extensively researched over recent decades, resulting in the intensive clinical testing now published in numerous multicentre phase I/II clinical trials [5] [6] [7] [8]. Initial Phase I monotherapy studies established reovirus as a safe clinical agent, although with insufficient anti-tumor activity to make it a successful stand-alone therapy [9] [10] [11]. However, recent pre-clinical and clinical testing has produced significant evidence showing a higher efficacy for reovirus when combined with traditional chemotherapeutic agents such as cisplatin [12], docetaxel [13] and as a triple combination with cisplatin and paclitaxel [14]. More recently, reovirus has also been shown to have enhanced activity when combined with BRAF and MEK inhibitors in melanoma [15].

The potential of combining reovirus with RT was initially described in *in vitro* and *in vivo* pre-clinical studies [16]. This work showed that the combination effect was particularly significant in cell lines which were relatively resistant to reovirus alone, although the mechanism of this enhanced effect was not fully elucidated. The study also assessed criteria relevant for clinical implementation of this combination therapy, showing that the virus itself was resistant to high doses of irradiation, ensuring that it would not be inactivated during treatment and that the enhanced cytotoxic effect was neither schedule- nor sequence-dependent. A subsequent Phase I clinical trial established that the combination of intratumoral reovirus and radiotherapy was tolerable and safe in patients with advanced cancers. There was also evidence of local efficacy and response in distant unirradiated disease [17].

In the current study, we build on the existing evidence and investigate the mechanism underlying the enhanced therapeutic efficacy of combining reovirus and RT in melanoma. Our data suggest that enhanced cytotoxicity is mediated through increased viral replication and activation of mitochondrial apoptotic signalling.

## RESULTS

### RT3D and RT combination shows synergistic cytotoxicity in melanoma

**Figure 1:**
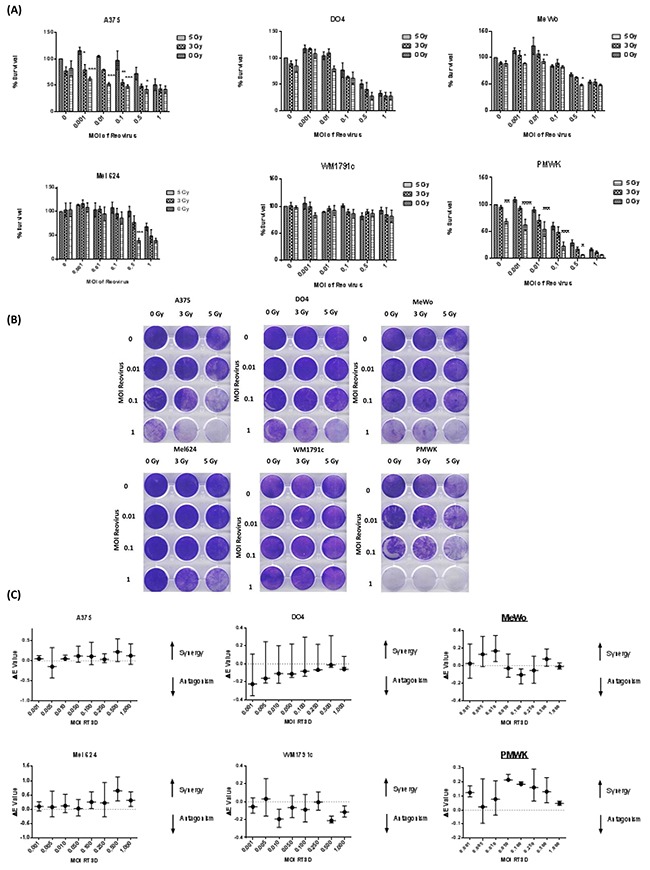
RT3D and RT combination in a panel of melanoma cell lines Cells were irradiated at either 3 or 5 Gy and 4 hours later infected with RT3D at a range of MOIs. Cell survival was measured by MTT **A.** and confirmed by crystal violet assay **B.** Synergy was assessed using Bliss analysis **C.**

The effect of RT3D and RT combination therapy was assessed in multiple melanoma cell lines of various genetic backgrounds, including ^V600E^BRAF mutant, Ras (K- and N-) mutant and BRAF/Ras wild-type (WT). Cells were either treated with RT3D alone or irradiated with either 3 or 5 Gy fractions and 4 hours later infected with RT3D at a range of MOIs (multiplicity of infection). The effects of both monotherapy and combination therapies were assessed 72 hours later by both MTT and crystal violet assay (Figure 1A, 1B). The N-Ras mutant DO4 cell line displayed high levels of sensitivity to the virus alone, compared to the K-Ras mutant cell line, WM1791c, which was resistant to the virus both as monotherapy or in combination with RT. Both ^V600E^BRAF mutant and WT cell lines displayed significantly enhanced cytotoxicity in the combination therapy groups; with ^V600E^BRAF mutant A375 and WT PMWK cell lines the most sensitive to the combination therapy, especially at the higher dose (5 Gy) of irradiation. To evaluate the level of synergy between RT3D and RT, we used Bliss independence analysis. The results from the Bliss analysis showed no synergy in either of the Ras mutant cell lines. However both BRAF mutant (A375 and Mel624) and WT (PMWK and MeWo) cell lines displayed a strong synergistic effect (Figure 1C).

### RT3D and RT combination therapy enhances cytotoxicity through mitochondrial apoptotic signalling

**Figure 2:**
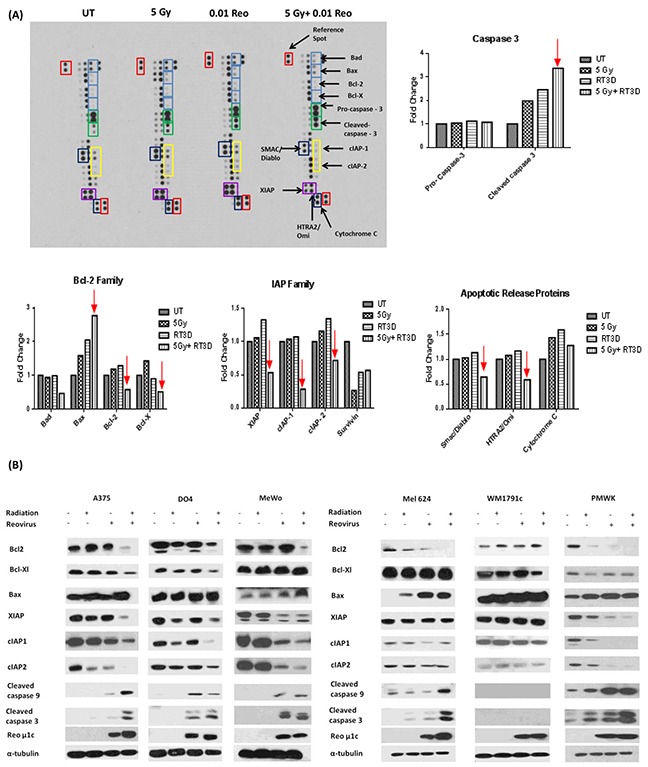
RT3D and RT combination therapy increases apoptosis in melanoma cells Human apoptosis array was used to assess expression levels of pro - and anti – apoptotic proteins in A375 cells. Cells were either untreated, infected with RT3D (MOI 0.1), irradiated (5 Gy) or treated with a combination of RT3D (MOI 0.1) and 5 Gy irradiation. Array membranes were analysed using Image J to determine differences in expression levels of proteins **A.** Results from array were confirmed by western blotting and expression was also assessed in the complete panel of melanoma cells **B.**

A human apoptosis array was carried out in the BRAF mutant A375 cell line 72hrs after treatment under the following conditions: untreated; 5 Gy irradiation; RT3D at MOI 0.01; and 5 Gy + RT3D at MOI 0.01 (Figure 2A). Densitometry analysis was carried out on the resulting images using Image J software and differences in the intensity of each antibody under each condition was graphed (Figure 2A). Analysis of the array showed a strong increase in the expression of cleaved caspase 3 in the combination group compared to either virus or RT alone. Enhanced caspase 3 cleavage was confirmed in the A375 cell line by western blot, with a similar effect also observed in the second BRAF mutant cell line Mel624 and the WT cell line PMWK (Figure 2B).

The combination therapy clearly displayed a predilection for modulating mitochondrial apoptotic signalling (graphs and western blots of extrinsic apoptotic signalling are shown in Supplementary Figure S1A, S1B). The combination therapy had a clear effect on the Bcl2 family of proteins, by down-regulating pro-survival proteins, such as Bcl2 and Bcl-XL, while up-regulating the pro-apoptotic protein Bax (Figure 2A). Western blot analysis also indicated the down-regulation of pro-survival Bcl2 and Bcl-XL and the up-regulation of pro-apoptotic Bax (Figure 2B).

The array also revealed down-regulation of the inhibitor of apoptosis (IAP) family of proteins which inactivate caspases. Western blot confirmed a decrease in XIAP, cIAP1 and cIAP2, observed in multiple cell lines, with a corresponding increase in cleaved caspase 9 and caspase 3 (Figure 2B).

Surprisingly, the array data seemed to indicate a decrease in the levels of mitochondrial pro-apoptotic proteins such as Smac/Diablo and cytochrome c (Figure 2A). We subsequently used confocal imaging and mitochondria/cytoplasm fractionation in A375 cells to determine if cytochrome c and Smac/Diablo were released from the mitochondria (Supplementary Figure S1C, S1D). Our results suggest that treatment with RT3D alone can release cytochrome c and Smac/Diablo from the mitochondria. While this may be enhanced by the addition of RT the effect appears to be primarily a viral response.

### RT enhances RT3D viral replication by inhibiting PKR activation mediated by CUG2

**Figure 3:**
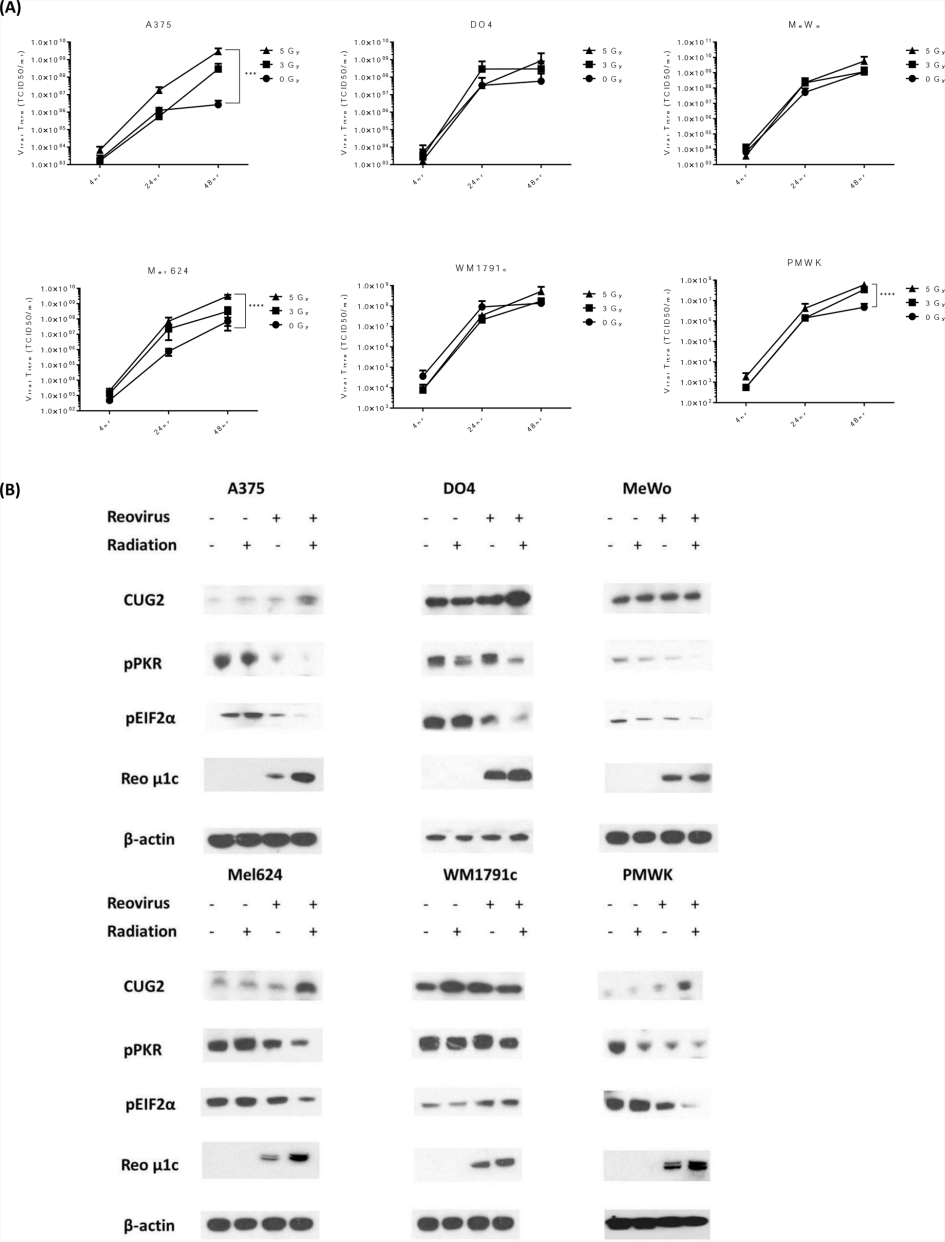
RT increases RT3D viral replication in melanoma cells through the suppression of PKR activation Cells were irradiated at either 3 or 5 Gy and 4 hours later infected with RT3D (MOI 5). The cells were harvested and supernatant was collected at 4, 24 and 48hr after infection. Viral titres were assessed using one - step growth curve assays **A.** Cells were irradiated at 5 Gy and 4 hours later infected with RT3D (MOI 5). Expression of CUG2, pPKR, p-eIF2α and viral protein μ1c were detected by western blotting at 48hr **B.**

Viral replication was measured by both one-step growth curves and plaque assays. Cells were irradiated at either 3 or 5 Gy fractions and 4 hours later infected with RT3D at MOI of 5. Viral replication was assessed at 4, 24 and 48 hours by one-step growth curves (Figure 3A) and at 48 hours by viral plaque assay (Supplementary Figure S2). Both the growth curves and plaque assays showed a clear increase in replication for all cell lines, with the exception of the K-Ras mutant WM1791c cell line. There was a clear increase in replication for the BRAF mutant cell lines, A375 and Mel624, at both 24 and 48 hours in the viral growth curves and the plaque assays at 48 hours. The WT cell lines, PMWK and MeWo, also showed a significant increase in replication, particularly at 48 hours, on both assays (Figure 3A).

In order to determine how RT was leading to an increase in RT3D viral replication, we examined the expression of proteins known to play a role in RT3D viral replication. Increased expression of cancer up-regulated gene 2 (CUG2) has previously been reported to enhance RT3D viral replication [18]. Upregulation of CUG2 was observed for both BRAF mutant cell lines and the WT PMWK cell line following RT3D and RT. This was associated with a corresponding decrease in pPKR and p-eIF2α expression, allowing for the enhanced translation of viral proteins as demonstrated by increased expression of reoviral outer capsid protein, μ1c (Figure 3B). The N-Ras mutant DO4 cell line also showed an increase in CUG2 and downstream decrease in pPKR and p-eIF2α, although the effect was less pronounced. In the K-Ras mutant cell line WM1791c, consistent with the viral replication data, the combination therapy did not upregulate CUG2 and both PKR and eIF2α remained highly phosphorylated (Figure 3B). The WT MeWo cell line did not show an increase in CUG2. However PKR activation was inhibited in this cell line as levels of pPKR and p-eIF2α were decreased. This suggests that, in MeWo cells at least, PKR inactivation may be mediated by a protein other than CUG2.

### RT-induced RT3D viral replication is reversed by the inhibition of either Ras or p38

**Figure 4:**
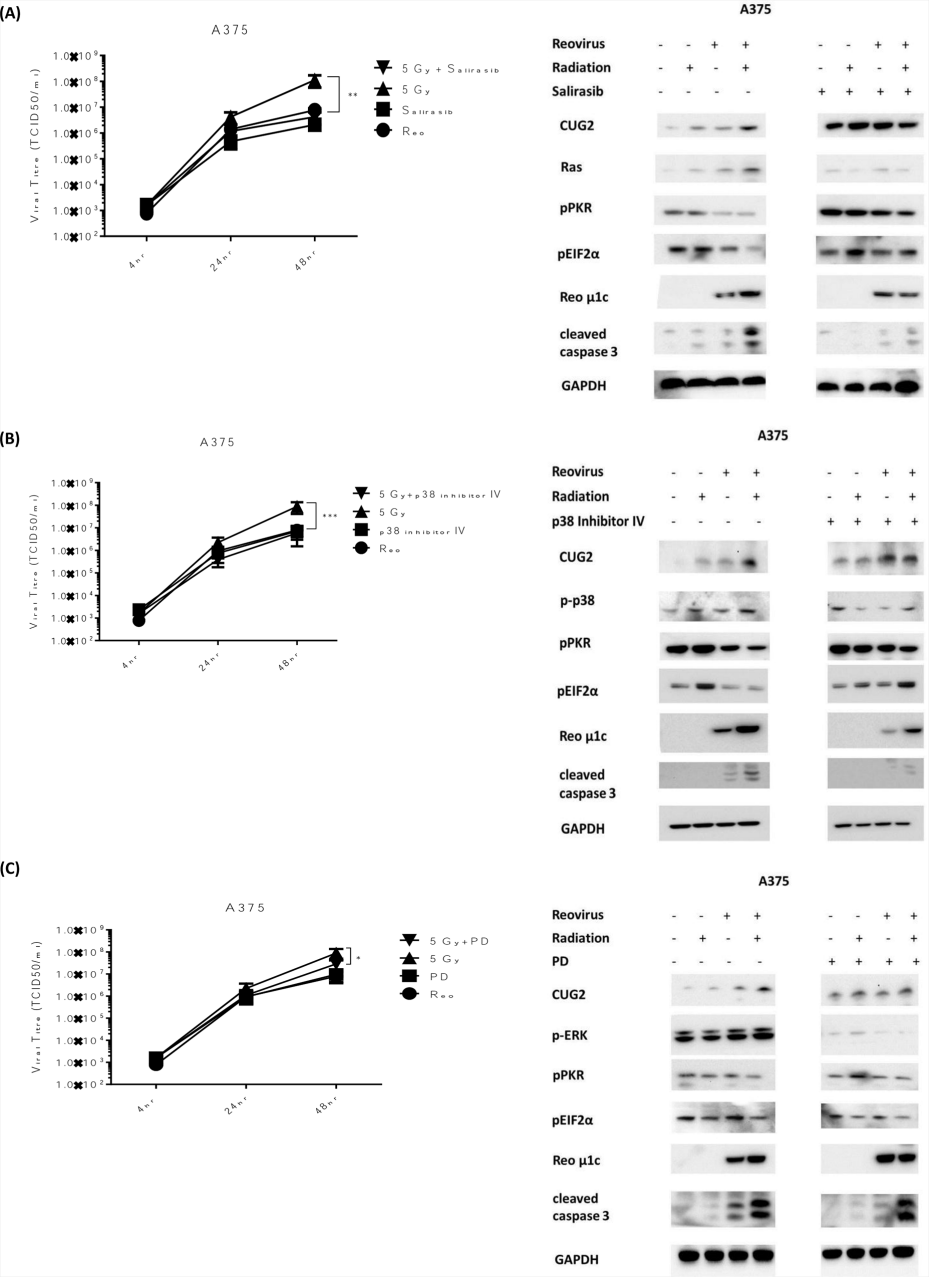
RT-induced viral replication is reversed by inhibition of either Ras or p38 A375 cells were treated with salirasib (10 μM) **A.**, p38 inhibitor IV (10 μM) **B.** or PD184352 (1 μM) **C.**, 1hr before irradiation at 5 Gy and 5 hrs before infection with RT3D (MOI 5). Viral titres were assessed by one - step growth curve assays (A-C). Protein was isolated at 48hrs and expression of CUG2, pPKR, p-eIF2α, cleaved caspase 3 and viral protein μ1c were detected by western blotting (A-C). Expression of Ras (A), p-p38 (B) and pERK (C) were also assessed.

The initial study proposing a role for CUG2 in reoviral replication [18] determined that CUG2 worked through the activation of Ras and p38 signalling. We, therefore, hypothesized that if RT was enhancing replication through a CUG2-Ras-p38 signalling cascade inhibition of either Ras or p38 should prevent RT-induced viral replication. We also hypothesized that, in line with the initial study, inhibition of other MAPK proteins, such as ERK, should not greatly affect RT-induced replication. BRAF mutant A375 cells were used in this experiment. Cells were pre-treated with either salirasib (10 μM), p38 inhibitor IV (10 μM) or PD184352 (1 μM) 1 hour before irradiation at 5 Gy. Cells were then infected with RT3D (MOI 5) 4 hours after irradiation, consistent with previous replication assays. Replication was measured by one-step growth curve assays. Protein was isolated under the same conditions at 48 hours for western blot analysis. Pre-treatment of cells with either salirasib or p38 inhibitor IV significantly reduced RT-induced replication returning it to baseline levels (Figure 4A and 4B). Pre-treatment with the PD compound also had an effect on RT-induced replication, albeit to a much lesser extent compared to either of the other inhibitors (Figure 4C). In correlation with the replication data, both pPKR and p-eIF2α showed consistently high phosphorylation levels in the combination group when pre-treated with either Salirasib or p38 inhibitor IV. The pre-treated combination group for these two inhibitors also displayed lower expression of both viral μ1c and cleaved caspase 3, indicating a link between increased viral replication and cell death (Figure 4A and 4B). Pre-treatment with the PD compound, however, appeared to have little effect on the phosphorylation levels of both PKR and eIF2α or the high levels of μ1c and cleaved caspase 3, despite its effects on viral replication (Figure 4C). Taken together these results support previous reports that signalling through Ras and p38, but not ERK, is needed for CUG2-associated viral replication.

### Inhibition of RT3D viral replication decreases apoptotic signalling

**Figure 5:**
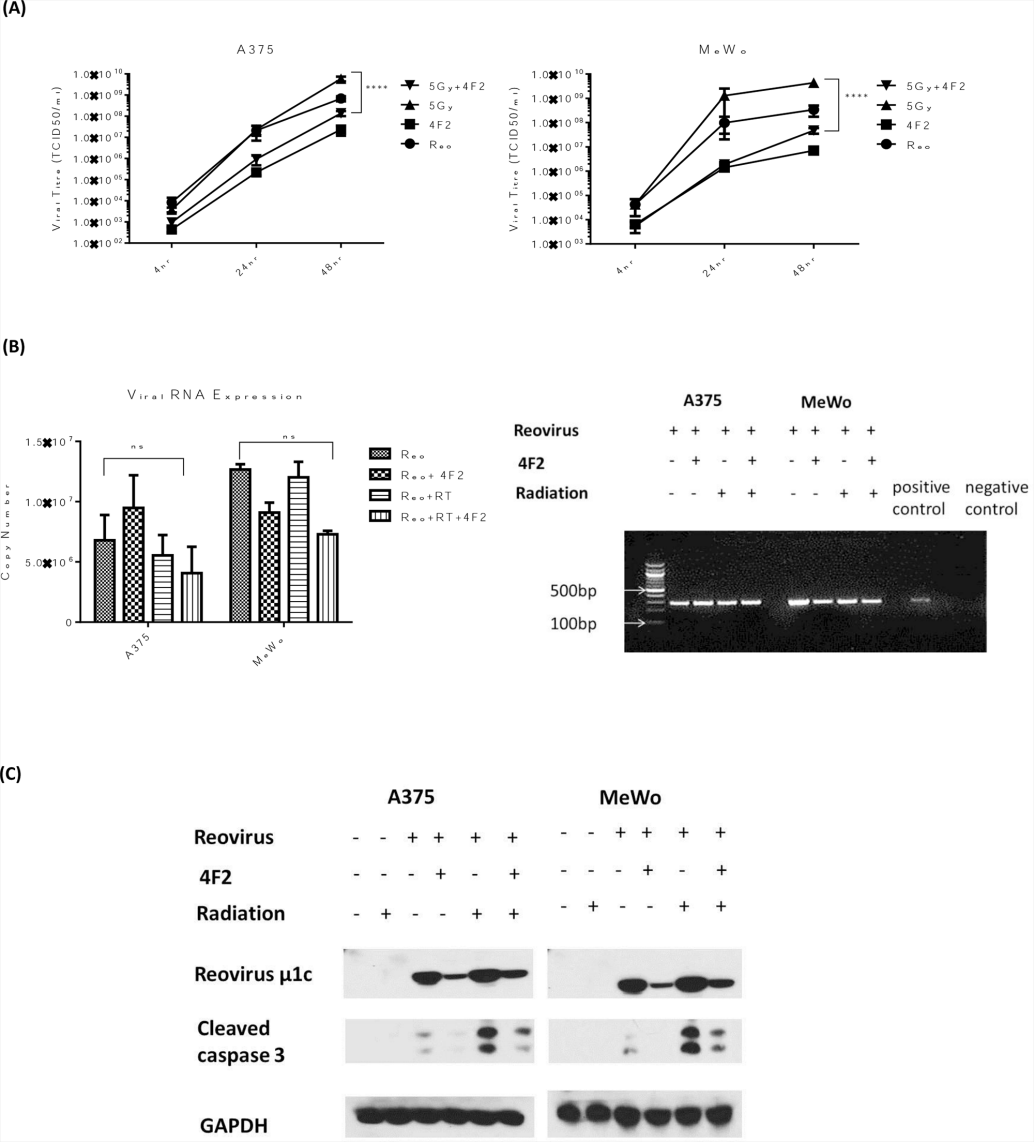
Inhibition of RT3D viral replication decreases apoptotic signalling RT3D was pre-incubated with 4F2 antibody overnight at 4°C. A375 and MeWo cell lines were irradiated at 5 Gy and 4 hours later infected with either RT3D (MOI 5) or RT3D + 4F2 antibody (MOI 5). The cells were harvested at 4, 24 and 48hr after infection and viral titres assayed by one - step growth curve assay **A.** Viral RNA was isolated from the 4 hr time point and analysed by both qPCR and a one – step PCR assay. PCR products were visualized on an agarose gel **B.** Protein was isolated at 48hrs and expression of cleaved caspase 3 was detected by western blotting **C.**

To explore the link between replication and apoptosis, replication was curtailed by binding the virus to the monoclonal antibody 4F2 (which targets sigma 3 protein), before infection of cells. Previous studies [19, 20] established that 4F2 inhibits the production of virus particles by blocking virus replication. Viral replication was measured by one-step growth curves in A375 and MeWo (BRAF mutant and WT, respectively) cell lines. The effect of radiotherapy on replication was diminished in both cell lines when the virus was pre-incubated with 4F2 antibody (Figure 5A). In order to ensure that the decrease in replication was not due to the 4F2 antibody blocking viral entry into the cell, both qPCR and a one-step PCR assay were carried out using RNA collected from the 4hr time point. Pre-incubation with 4F2 antibody did lower the copy number for the RT sample in both cell lines however the difference was not significant. PCR products from the one-step assay, visualized on an agarose gel, showed strong bands in all the samples. Both these assays indicated that the antibody did not prevent viral entry into the cell (Figure 5B). Analysis of cleaved caspase 3 signalling indicated that there was also a decrease in the level of apoptosis across the cell lines when the virus was pre-incubated with 4F2 antibody (Figure 5C). These results suggest that the increase in replication and enhanced apoptosis do not occur independently of each other, but rather are linked with the enhanced replication promoting apoptosis.

### Combined RT3D and RT therapy *in vivo* prolongs survival in A375 ^V600E^BRAF mutant xenograft tumours

**Figure 6:**
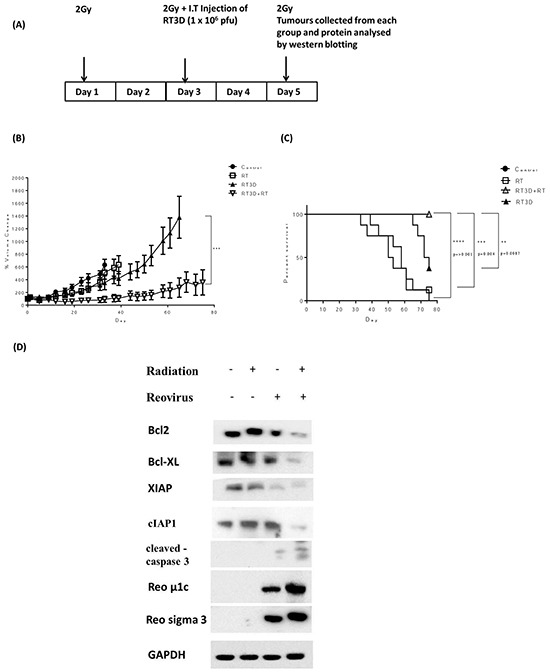
Combined RT3D and RT therapy enhances tumour reduction in ^V600E^BRAF mutant melanoma *in vivo* A375 subcutaneous xenograft tumours were established in CD1 nude mice. Mice were either untreated, irradiated with 2 Gy fractions for 3 cycles, RT3D infected at 1 × 10^6^ pfu/ml or combined treatment (2 Gy fraction for 3 cycles and 1 × 10^6^ pfu/mL RT3D) **A.** The size of tumours was measured for each treatment group and the % volume change for each group plotted. Each bar represents ±SEM of at least 8 mice. **B.** A Kaplan-Meier curve was used to assess median survival rate for each treatment group. **C.** Tumours were collected from one mouse per cohort on day 5 of treatment and levels of apoptotic proteins assessed by western blot. Protein was isolated from tumours and expression of Bcl2, Bcl-XL, XIAP, cIAP1, cleaved caspase 3, μ1c and sigma 3 were detected by western blotting **D.**

As a result of the potential therapeutic efficacy of combining RT3D and RT observed in the *in vitro* study, we assessed the therapeutic potential of this combination *in vivo*. A375 xenograft tumours were established in CD1 nude mice. Mice were randomly allocated into the following groups: untreated; RT3D alone at 1 × 10^6^ plaque forming units (PFU); RT alone (6 Gy in three fractions over 5 days); and a combination of 6 Gy in three fractions plus 1 × 10^6^ PFU RT3D. The treatment timeline is summarised in Figure 6A. One mouse from each group was culled at the end of treatment on day 5 for pharmacodynamics analysis. Tumour volume from the remaining mice in each group was measured and % volume change was plotted to day 80 after treatment (Figure 6B). While RT3D monotherapy did delay tumour growth and prolonged survival compared to RT monotherapy, the combination therapy was significantly more effective than either monotherapy at delaying tumour growth. Survival analysis for the combination cohort also showed that these mice had a lower mortality rate compared to either monotherapy or control cohorts (Figure 6C). Consistent with *in vitro* data, western analysis showed enhanced mitochondrial apoptotic signalling in the combination group resulting in increased caspase 3 cleavage. An increase in the expression of viral outer capsid proteins μ1c and sigma 3 indicated enhanced viral replication - also consistent with *in vitro* data (Figure 6D).

## DISCUSSION

Melanoma is one of the deadliest forms on skin cancer and is on the rise worldwide. A major obstacle in the treatment of malignant melanoma is its resistance to classical chemotherapy and radiotherapy, which may be due, in part, to a natural resistance to apoptosis [21] attributed to high levels of anti-apoptotic Bcl2 proteins [22]. In recent years, much progress has been made in understanding the genetic background of this disease and identifying specific targets for drug development. For instance, Ras/Raf/Mek signalling has come to the fore with the finding that up to 50% of melanomas have a BRAF mutation and up to 30% have a NRAS mutation [23] [24, 25], with resulting inhibitors targeting BRAF specifically [26] and MEK signalling in general [27].

Aside from traditional drug development strategies, other areas of research have focussed on the development of biological therapies including the use of oncolytic virus therapy. Numerous pre-clinical and clinical studies have been carried out using a diverse range of oncolytic viruses including vaccinia [28, 29], HSV [30], VSV [31] and reovirus [32], both as monotherapies or in combination with traditional anti-cancer agents. The recent successful phase III clinical trial of HSV [33] in melanoma and the subsequent FDA approval of the virus have highlighted the potential of oncolytic viruses as anti-cancer agents, especially in melanoma.

Utilizing our growing understanding of this disease, we assessed the therapeutic potential of combining RT3D and RT in a panel of melanoma cell lines which included BRAF mutant, Ras mutant and BRAF/Ras wild-type cells. Previous work carried out in our lab combining GLV-1h68 (oncolytic vaccinia virus) and RT showed a synergistic effect in BRAF mutant melanoma that is mediated through JNK and TNFα signalling [34]. In this current study, while a synergistic effect was observed in BRAF mutant cells, it was also present in the BRAF/Ras wild-type cells, indicating that any increased effect was independent of Ras/Raf genotype (Figure 1A–1C). This synergy indicated a potential therapeutic advantage for the combination therapy and so we explored the mechanism behind our observations.

Cell death can occur through various mechanisms, with reovirus having previously been shown to induce apoptosis by both the death receptor signalling pathway, mediated by TRAIL, and the intrinsic mitochondrial pathway [35–37]. We assessed apoptotic signalling in a panel of cell lines to determine which mechanism was in play. An apoptosis array carried out on BRAF mutant cells indicated that signalling was through the intrinsic mitochondrial apoptotic pathway rather than through receptor-mediated extrinsic signalling (Figure 2A, 2B). This result was confirmed across the panel of cell lines, with decreases in pro-survival and corresponding increases in pro-apoptotic Bcl2 proteins observed in the combination group. Co-ordinate decreases in the expression of inhibitor of apoptosis proteins and increases in both cleaved caspase 9 and cleaved caspase 3 reinforced the argument that amplification of mitochondrial apoptotic signalling was the primary mechanism of cell death for the combination of RT3D and RT (Figure 2C).

Apoptosis can be triggered by various stress stimuli within the cell, in the case of invading viral particles an increase in viral replication is a widely accepted method of viral cell kill [13, 38]. While this does not always prove to be the case [39, 40], we hypothesized that the combination of RT and RT3D would result in an increase in viral replication, ultimately resulting in cell death. Our data clearly validated this hypothesis, with both one–step growth curves and plaque assays displaying a significant increase in viral replication with the addition of RT (Figure 3A, Supplementary Figure S2). When the virus was prevented from replication by pre-incubation with a sigma 3 antibody (4F2), we observed a decrease in cleaved caspase 3 signalling indicating reduced levels of apoptosis (Figure 5). The protective properties of various anti-reovirus monoclonal antibodies was first proposed by Virgin *et al* [20] who showed that these antibodies could inhibit the replication process at various stages, including binding to the cell, internalization or uncoating of the viral particle inside the cell. The sigma 3 antibody used in this study was found to act after the virus particle was bound to the cell and either at or before the un-coating of the viron. Therefore, for our study, equivalent amounts of virus particle were able to enter the cell regardless of the presence or absence of antibody, which was confirmed by PCR 4 hours after infection (Figure 5B). We can conclude, therefore, that decreased viral replication and subsequent decrease in apoptosis was not an effect of viral entry.

In order to confirm the therapeutic potential of RT and RT3D combination therapy, an *in vivo* study was carried out using a BRAF mutant A375 xenograft model. The *in vivo* results confirmed our *in vitro* observations with a significant increase in survival in the combination group and also the lowest percentage tumour volume change compared to the control group or either monotherapy group (Figure 6). Analysis of tumours isolated on day 5 of the treatment regimen confirmed the activation of mitochondrial apoptotic signalling with a decrease in both pro-apoptotic Bcl2 proteins and IAP proteins, as well as an increase in cleaved caspase 3 proteins. An observed upregulation of reovirus protein μ1c in the combination group also indicated an increase in replication, again confirming the *in vitro* findings.

**Figure 7:**
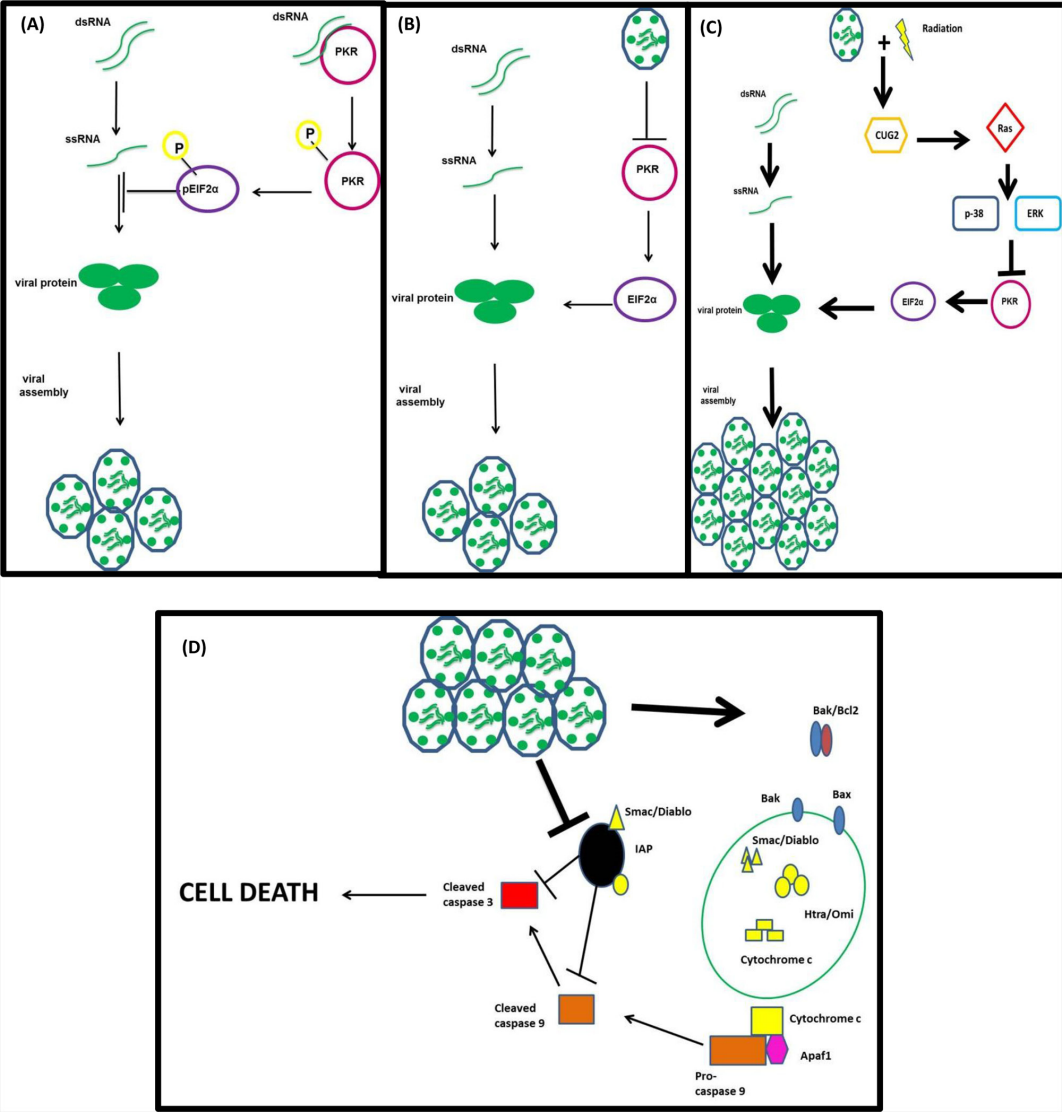
Proposed mechanism of action of RT3D + RT combination therapy The PKR recognizes and binds to double-stranded RNA molecules resulting in its auto-phosphorylation and the subsequent downstream phosphorylation of eIF2α. The phosphorylation of eIF2α switches off the host cell's translational machinery preventing viral protein translation **A.** RT3D prevents the phosphorylation of PKR leaving eIF2α in an un-phosphorylated state and the host cell's translational machinery intact **B.** RT combined with up-regulates CUG2 resulting in the activation of Ras and the downstream protein p38. The activation of this pathway switches off the PKR defence mechanism, allowing viral protein translation and increasing viral replication **C.** The increase in viral replication ultimately results in cell death via mitochondrial apoptotic signalling **D.**

The logical question arising from these results is; how does RT contribute to the increase in RT3D replication? To answer this question, we decided to probe the host cell's anti-viral defence mechanisms, in particular the activation of the PKR pathway. PKR is a protein that recognises and binds to double-stranded RNA molecules, resulting in auto-phosphorylation of the protein. This phosphorylated PKR then phosphorylates eIF2α, which inhibits the host cell's translational machinery preventing the translation of viral proteins (Figure 7A). A recent study by Park *et al* [18] showed that this pathway could be deactivated by the overexpression of the oncogene CUG2, through activation of the MAPK pathway, specifically Ras and p38, resulting in an increase in reovirus replication (Figure 7B). When we examined this signalling pathway in our panel of cell lines, there was evidence of activation in the combination group in the majority of cell lines. Both BRAF mutant and WT cell lines, which displayed the most significant RT-induced increase in replication (Figure 3), showed up-regulation of CUG2 in the combination group and a deactivation of the host's anti-viral defences by decreased levels of both pPKR and p-eIF2α (Figure 3).

Although the up-regulation of CUG2 in the combination group implicated this pathway in RT-induced viral replication, further confirmation was needed. As previous reports suggested a role for MAPK activity in connection with CUG2 signalling, inhibitors of Ras, p38 and ERK were used to determine if the increase in replication could be reversed. As shown in Figure 4, inhibitors of either Ras (salirasib) or p38 (p38 inhibitor IV) significantly reduced the effect of RT on viral replication, returning levels to baseline. Inhibition of ERK, however, did not have the same dramatic effect, supporting previous work indicating a role for Ras and p38, but no other MAPK proteins, in CUG2-mediated reovirus replication (Figure 4).

Since it was first identified as an oncogene in 2007 [41], CUG2 overexpression has been linked to apoptosis in ovarian cancer cells [42] and migration and drug resistance in colon cancer cells [43]. As it has also been shown to predominantly localize to the nucleus [44], there is no clear mechanism of cross-talk between CUG2 and the activation of the pathways mentioned above. The previous report linking CUG2 and Ras to reovirus replication did not show if Ras activated CUG2 or *vice versa*. In our study, the inhibition of Ras by salirasib did not reduce the levels of CUG2, indicating that Ras does not induce CUG2 expression (Figure 4A). Although our data provide further evidence for the role of CUG2, Ras and p38 in reovirus replication, the precise mechanism of cross-talk within this signalling pathway remains to be fully elucidated.

Switching off the PKR pathway is an important viral tactic in breaching the host cell's defences; however it is by no means the only weapon at its disposal. Recent work has highlighted the role of PTEN in anti-viral innate immunity by activating an interferon response. Specifically PTEN was found to control the import of the transcription factor IRF3 into the nucleus where it activates interferon –responsive genes [45] [46]. Li *et al* showed that PTEN deficient models, both *in vitro* and *in vivo,* were more susceptible to the oncolytic virus VSV due to a decrease in the induction of type 1 interferon reducing the anti-viral immune response. In an era of increased interest in understanding the complex interaction between the immune system and tumor cells it is possible that combining novel biological therapies with traditional agents will unlock new avenues of immune activation which can be manipulated to deliver a more targeted therapy and improve patient outcomes.

Reovirus is currently being tested as an anti-cancer therapy in phase I/II clinical trials. Previous studies, both pre-clinical and clinical, have shown that reovirus is more effective as a combination agent rather than a stand-alone therapy. Our research provides further evidence of the therapeutic potential of this agent and builds on previously published studies highlighting the benefits of combining this oncolytic virus with radiotherapy.

## MATERIALS AND METHODS

### Cell lines

The following cell lines (obtained from Prof. Richard Marais) were used: A375, Mel624, (^V600E^BRAF mutant), MeWo and PWMK (wild-type RAS and BRAF), D04 (^Q61L^N-RAS mutant), WM17971c (^Q61H^K-RAS mutant) and L929 mouse fibroblasts (Oncolytics Biotech, Inc.). D04 and WM1791c cells were cultured in RPMI (Roswell Park Memorial Institute) media, all other cells were cultured in DMEM (Dulbecco's Modified Eagle's Medium). Media were supplemented with 5% (v/v) FCS, 1% (v/v) glutamine, and 0.5% (v/v) penicillin/streptomycin.

### Reovirus stocks

Reovirus Dearing type 3 (RT3D) stocks at 2.8 × 10^9^ tissue culture infectious dose 50 (TCID_50_/ml) were obtained from Oncolytics Biotech and stored at 1:10 concentrations either in PBS or DMEM containing 5% (v/v) FCS, 1% (v/v) glutamine, and 0.5% (v/v) penicillin/streptomycin at −80°C.

### 3-(4, 5-dimethylthiazol-2-yl)-2, 5-diphenyltetrazolium bromide assay

Cell viability was quantified using a 3-(4, 5-dimethylthiazol-2-yl)-2, 5-diphenyltetrazolium bromide (MTT) assay. 20 mL MTT (thiazolyl blue; Sigma-Aldrich) at 5 mg/mL in PBS was added to treated cells in a 96-well plate for 4 hours at 37°C. Crystals were solubilised in DMSO and absorbance measured at 570 nm on a SpectraMax 384 plate reader (Molecular Devices).

### Crystal violet survival assay

Cell viability was assessed by crystal violet staining (Sigma-Aldrich) at 0.2% (w/v) in a 7% (v/v) solution of ethanol/PBS. Plates were incubated at room temperature for 1 hour and then washed twice in distilled water. The crystal violet stained images of the plate were captured on a Microtek ScanMaker 8700 (Microtek International Ltd).

### Replication assays

Melanoma cells were seeded in 24-well plates at a density of 1 × 10^5^ cells/well. Culture plates were then irradiated at either 3 Gy or 5 Gy the next day and infected with RT3D at an MOI of 5 for 2 hours. The cells were washed twice in complete growth media. Complete growth media was added to the cells and incubated at 37^°^C. The cells were harvested and the supernatants were collected at 4, 24, and 48 hours post-infection in triplicate. The lysates had three freeze-thaw cycles between −80°C and room temperature. For one-step growth curves the resulting lysates were titrated on L929 cells in 96-well plates. Viral titers were determined using the TCID_50_/method as previously described [16]. For plaque assays, dilutions were used to infect L929 cells seeded at 2 × 10^5^ cells/well in 6 well plates. After incubation at 37^°^C for 4 hours, the viral medium was removed and the wells overlaid with a 1:1 solution of 2% agar (Sigma) and 2X DMEM containing 5% (v/v) FCS, 1% (v/v) glutamine, and 0.5% (v/v) penicillin/streptomycin. After 5 days, plates were stained with 0.2% crystal violet in 7% ethanol. Plates containing plaques were scanned and counted using OpenCFU software [47].

### PCR

Viral RNA was extracted from the 4 hour replication time point using a Qiagen QIAamp Viral RNA kit. RNA isolated from stocks was used as a positive control. One step RT-PCR was then carried out using Qiagen One Step RT-PCR kit with the following primers; forward 5′GGGCTGCACATTACCACTGA 3′, reverse 5′CTCCTCGCAATACAACTCGT 3′. PCR products were then resolved on a 2% agarose gel, stained with EtBr (Sigma) and visualized using a UV trans-illuminator. Quantitative PCR was carried out on viral RNA samples using Bioline SensiFAST™ SYBR® Hi-ROX One-Step Kit. The following primers were used; forward 5′ TTAGGATGGCCTCGTCCTTT 3′, reverse 5′TTCATCATCACGCAAAACCT 3′. A standard curve was created using viral stock RNA. Copy number was calculated using online copy number calculator (http://scienceprimer.com/copy-number-calculator-for-realtime-pcr). All qPCR assays were carried out using Step One™ Real-Time PCR System (Applied Biosystems).

### Inhibitor assays

For replication assays, cells were seeded in 24-well plates at 1 × 10^5^ cells/well and for western blotting in 60 mm dishes at 2 × 10^5^ cells/dish. Plates were then treated with one of the following inhibitors; salirasib [10 μM] (Sigma), p38 inhibitor IV [10 μM] (Sigma) or PD 184352 [1 μM] (Selleck), 1 hour before irradiation at 5 Gy. Cells were infected with RT3D (MOI 5) 4 hours after irradiation. One-step growth curves and western blotting were carried out as described.

### Confocal imaging

Cells were seeded at 1 × 10^5^ cells/dish, irradiated with 5 Gy the next day and infected with RT3D (MOI 0.01). Cells were incubated at 37^°^C for 48 hours, and then 100 nM Mitotracker (Cell signalling) was added to each plate and incubated at 37^°^C for 15 min. The Mitotracker was removed and replaced with fresh growth medium and incubated at 37^°^C for 1 hour. Cells were fixed in 4% paraformaldehyde (PFA) at 37°C for 10 min, washed in PBS (3 × 5 mins), permeabilized with 0.2% Triton X-100 (Sigma) in PBS for 20 min and washed in PBS 3 times. Cells were then blocked in immunofluorescence buffer (IFF; PBS, 1% BSA, 2% FBS and 0.05% sodium azide) for 1 hour at room temperature and incubated overnight with primary antibody (1:200). Secondary Alexa fluor (1:1000 Life Technologies) was added and incubated at room temperature for 90 min. Cells were washed in PBS once for 5 min. Images were taken using a Zeiss Zen 2009 confocal system.

### Apoptosis array

Cell lysates were obtained from A375 cells, after 72hrs post-treatment. The expression of 35 apoptosis-related proteins was analysed using the antibody-based Proteome Profiler™ Apoptosis Array from R&D Systems (Cat No. ARY009) as per manufacturer's instructions.

### Western blotting

Cells were plated at 2 × 10^5^ in 60 mm dishes. Following various treatments, cells were harvested in ice-cold PBS, pelleted and re-suspended in radioimmunoprecipitation assay buffer [50 mM Tris (pH 7.5), 150 mM NaCl, 1% NP40, 0.5% sodium deoxycholate, and 0.1% SDS] with protease inhibitors (Roche Diagnostics GmbH, Mannheim, Germany), 1 mM sodium orthovanadate (Sigma), and 10 mM sodium fluoride. Cells were then lysed by snap freezing on dry ice and allowing the lysate to thaw on ice for 10 minutes before centrifugation at 13,200 rpm/4°C for 20 minutes to remove cell debris. Protein concentration was determined using the BCA protein assay (Pierce, Rockford, IL). Twenty-five μg of each protein sample were resolved on SDS-polyacrylamide gels (10-12%) and transferred to a polyvinylidene difluoride Hybond-P membrane (Amersham, Buckinghamshire, United Kingdom). The membrane was blocked with 5% milk in Tris-buffered saline with Tween 20 at room temperature for 1 hour incubated overnight at 4°C with primary antibody. Primary antibodies for all proteins were obtained from Cell Signalling, except the following: smac/diablo (Abcam), pan Ras (biorybt), pPKR (Santa Cruz Biotechnologies), CUG2 (ProSciΨ™), reo μ1c 10F6 and sigma 3 4F2, (Developmental Studies Hybridoma Bank). Equal loading was assessed using a-tubulin (Sigma Aldrich), β-actin (Abcam) or GAPDH (Cell Signaling) mouse monoclonal primary antibodies. Blots were developed using secondary antibody conjugated to horseradish peroxidase (1:5000; GE Healthcare) and visualized using Super Signal chemiluminescent system (Pierce) or Immobilon Western chemiluminescent HRP substrate (Millipore).

### *In vivo* studies

A375 tumours were established in female CD1 nude mice by subcutaneous (s.c) injection of 3 × 10^6^ cells suspended in 100 μL PBS in the left flank. Once xenografts had reached approximately 5 mm in diameter, mice were randomly allocated to treatment groups (10 mice per group) before beginning therapy, prior to irradiation. Anesthetized animals (I.P. injection of 100 μL of a 1:10:20 mixture of ketamine (Vetalur 100mg/ml), xylazine (Rompin 2%) and water for injection BP (Fresenius Health Care Group)) were positioned with tumours exposed under an aperture in 3-mm lead shielding and irradiated for a total dose of 6 Gy in 3 fractions over 5 days. RT3D was administered by intra-tumoral injection 1 hour before the second fraction of radiation. Tumors were harvested from one mouse in each group after 5 days treatment. Tumours from the remaining mice were measured twice weekly in two dimensions using Vernier callipers and the volume estimated using the formula (length^2^ × width)/2. Humane endpoint was defined as a tumor diameter greater than 12 mm in any dimension.

### Statistical analysis

Comparisons between groups were done using the two-way ANOVA (in vitro analysis). P values <0.05 were considered to be statistically significant (*, *P* < 0.05; **, *P* < 0.01; ***, *P* < 0.005). All plots were generated using Prism Graph Pad software. Survival analysis was done using Prism Graph pad software and log-rank test was used to calculate p value.

The MTT data was analyzed for synergy between different treatments by Bliss Independence Analysis as described previously [34]. Briefly, the Bliss formulae is as follows; E_IND_ = E_A_ + E_B_ – E_A_ × E_B_ and ΔE = E_OBS_ – E_IND_; where E_A_ and E_B_ are the fractional effects of factors A and B (in this case RT3D and RT), E_IND_ is the expected effect of the independent combination of factors and E_OBS_ is the observed effect of the combination. The ΔE values were classified as follows; > 0 synergy present, < 0 antagonism present or = 0 the combination was independent.

## SUPPLEMENTARY FIGURES


