# Supplementary material for: Enhanced cytotoxicity of reovirus and radiotherapy in melanoma cells is mediated through increased viral replication and mitochondrial apoptotic signalling
Source: Oncotarget. 2016 Jul 1;7(30):48517–32. doi: 10.18632/oncotarget.10365 (PMC5217035; doi:10.18632/oncotarget.10365)
Supplement: Supplementary file 1 [file oncotarget-07-48517-s001.pdf]

Enhanced cytotoxicity of reovirus and radiotherapy in melanoma cells is mediated through increased viral replication and mitochondrial apoptotic signalling

SUPPLEMENTARY FIGURES

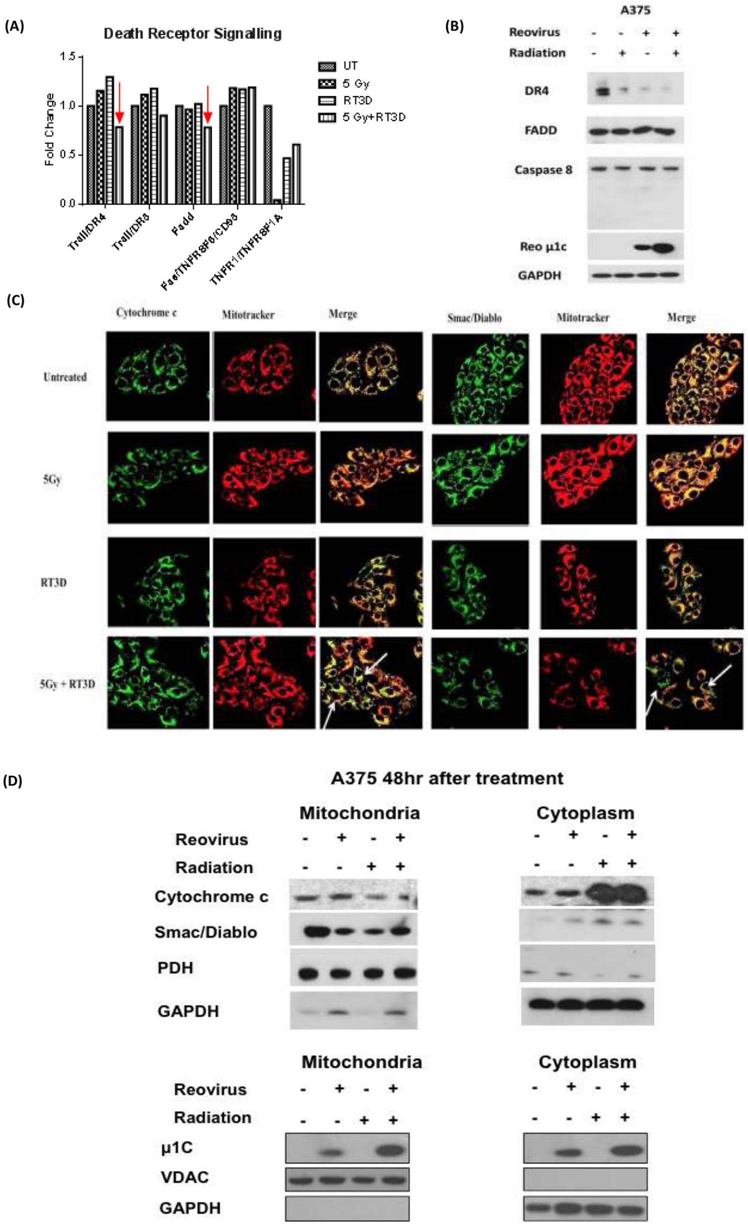

Supplementary Figure S1: RT3D and RT combination therapy does not activate extrinsic apoptotic signalling in BRAF mutant A375 cells. Densitometry analysis of human apoptosis array shows a general decrease in expression of extrinsic apoptosis in the combination group **A.** This result was confirmed by western blot analysis which showed no evidence of caspase 8 cleavage or activation of either death receptor 4 (DR4) or FADD in the combination group **B.** Confocal imaging was used to determine if cytochrome c and smac/diablo were released from the mitochondria. Protein present in the mitochondria appears yellow in the merged image while protein present in the cytoplasm remains green highlighted by the white arrows. The results indicate that RT3D alone is sufficient to release both proteins from the mitochondria **C.** This was confirmed by western blot analysis of fractionated mitochondria and cytoplasm samples. GAPDH was used as the cytoplasm loading control and pyruvate dehydrogenase (PDH) as the mitochondrial loading control **D.** (upper panel). m1c was assessed in the fractionated proteins and showed RT3D to be present in both the cytoplasm and mitochondria.

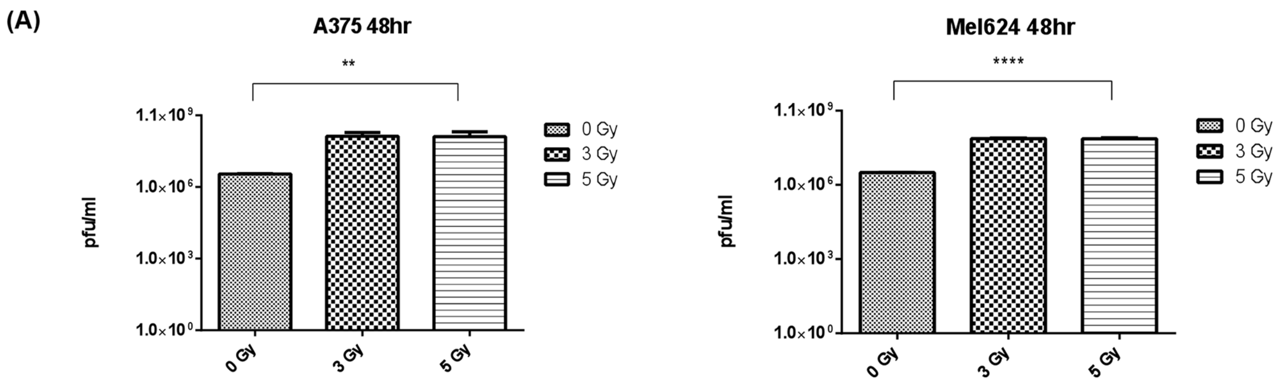

Supplementary Figure S2: The effect of RT on RT3D replication was also assessed by viral plaque assay. Cells were irradiated at either 3 or 5 Gy and 4 hours later infected with RT3D (MOI 5). Cells were harvested and supernatant collected at 48 hour after infection. Viral titres were assessed by plaque assay on L929 cells. There is an increase in replication across the cell lines with the exception of the K-Ras mutant WM1791c cell line consistent with one - step growth curve assay.
